# Application of three-dimensional Superb micro-vascular imaging (3D-SMI) combined with quantitative blood flow analysis in the noninvasive diagnosis of renal tumors

**DOI:** 10.1186/s13089-025-00464-y

**Published:** 2025-12-01

**Authors:** Yiran Mao, Chunyang Yu, Tianqi Wang, Fangxuan Li, Wenjing Hou, Xi Wei, Jie Mu

**Affiliations:** 1 Department of Ultrasound, Tianjin Medical University Cancer Institute and Hospital, Huanhuxi Road, Hexi District, Tianjin, 300060 China; 2 National Clinical Research Center for Cancer, Tianjin, China; 3 Key Laboratory of Cancer Prevention and Therapy, Tianjin, China; 4 Tianjin’s Clinical Research Center for Cancer, Tianjin, China; 5 Ultrasound Department, Peking University Binhai Hospital, Tianjin Fifth Central Hospital, Tianjin, China; 6 Department of Infectious Diseases, Tianjin Children’s Hospital, Tianjin, China; 7 Children’s Hospital, Tianjin University, Tianjin, China; 8 Department of Cancer Prevention, Tianjin Medical University Cancer Institute and Hospital, Tianjin, China

## Abstract

**Objective:**

To investigate the diagnostic value of three-dimensional superb micro-vascular imaging (3D-SMI) combined with quantitative analysis of Area and VI in differentiating benign and malignant renal tumors.

**Methods:**

A total of 256 renal lesions from 254 patients who underwent gray-scale ultrasound (Gray US), two-dimensional superb micro-vascular imaging (2D-SMI), and 3D-SMI examinations at Tianjin Medical University Cancer Institute and Hospital between January 2022 and June 2024 were retrospectively analyzed. The imaging features on Gray US, 2D-SMI and 3D-SMI were recorded. Based on 3D-SMI, Vascular Architecture were classified into five types: Type I (avascular), Type II (spotty flow), Type III (sparse flow), Type IV (encircling), and Type V (rich flow). The plane with the most abundant blood flow was selected, and the Area and VI were calculated using Image Pro Plus (IPP) software. Histopathology from surgery or biopsy served as the reference standard. The differences in Vascular Architecture, Area, and VI between benign and malignant renal tumors were compared, and their diagnostic performance was evaluated.

**Results:**

Among the 256 lesions, 70 were benign and 186 were malignant. The interobserver agreement for Vascular Architecture classification was good (Kappa = 0.803), and the consistency for Area and VI was high (ICC = 0.835 and 0.864, respectively). Benign tumors Vascular Architecture were mainly type II or III, with mean Area and VI values of 945.87 ± 568.26 (range: 68–3125) and 5.93 ± 4.95 (range: 0.23–24.73), respectively. Malignant tumors were predominantly type IV or V, with mean Area and VI values of 3694.53 ± 2612.38 (range: 93–9965) and 18.21 ± 10.83 (range: 0.69–48.13), respectively. Significant differences were observed in Vascular Architecture, Area, and VI between benign and malignant lesions (all *P* < 0.001). The area under the ROC curve (AUC) values for 3D-SMI Vascular Architecture, Area, VI, 2D-SMI, and Gray US were 0.813, 0.807, 0.859, 0.750, and 0.718, respectively. VI demonstrated the highest diagnostic performance, with a cutoff value of 8.19 (sensitivity: 82.26%; specificity: 85.51%). Among benign subtypes, there were no significant differences in Vascular Architecture or Area (*P* > 0.05), while the VI of oncocytoma was significantly higher than epithelioid angiomyolipoma (EMAL), metanephric adenomas (MA), and angiomyolipoma (AML)(*P* < 0.01). Among malignant subtypes, clear cell renal cell carcinoma (ccRCC) showed distinct Vascular Architecture compared with papillary renal cell carcinoma(pRCC), chromophobe renal cell carcinoma(chRCC), and Xp11.2 translocation/TFE3 fusion-associated renal cell carcinoma(tRCC) (*P* < 0.01). The Area and VI of ccRCC were significantly higher than those of pRCC and chRCC (*P* < 0.05), but not significantly different from tRCC (*P* > 0.05).

**Conclusion:**

3D-SMI provides three-dimensional visualization of Vascular Architecture. Quantitative analysis of the most vascularized plane using Area and VI differentiation between benign and malignant renal tumors, with VI demonstrating the best diagnostic efficacy. This technique offers a non-invasive diagnostic approach for renal tumors.

## Introduction

With the rapid advancement of imaging technology in recent years, the detection rate of asymptomatic renal masses has increased markedly. However, considerable overlap exists in the imaging features of benign and malignant renal tumors, making accurate preoperative characterization challenging [1]. Approximately 25% of renal masses smaller than 4 cm are misdiagnosed as malignant, leading to unnecessary surgical interventions and consequent risks such as renal function impairment [2]. Conversely, some malignant lesions with aggressive or metastatic potential remain undiagnosed in time, resulting in local progression, distant metastasis, and further renal damage [3]. Therefore, improving the accuracy of preoperative differentiation between benign and malignant renal tumors remains a critical challenge in clinical practice.

Ultrasound has become a widely used clinical imaging technique owing to its convenience, cost-effectiveness, and real-time capability. It provides valuable information regarding the size, location, echogenicity, and vascularity of renal lesions. However, conventional gray-scale and Doppler ultrasound still have limitations in qualitative diagnosis, often necessitating further contrast-enhanced evaluation [4].

Contrast-enhanced ultrasound (CEUS) has demonstrated diagnostic performance comparable to that of contrast-enhanced computed tomography (CECT) and contrast-enhanced magnetic resonance imaging (CE-MRI) in evaluating both cystic and solid renal lesions [5, 6]. Moreover, CEUS agent was purely intravascular, and non-nephrotoxic, making it suitable for patients with renal function impairment or Iodinated contrast agent allergy [7]. CECT can reflect histopathological features through enhancement patterns, but these findings are sometimes nonspecific, for instance, the central scar of oncocytoma may show hypoenhancement, is similar to necrotic areas in renal cell carcinoma [8]. CE-MRI offers superior spatial and contrast resolution and, through multiparametric imaging, can better depict histologic features, assess tumor cellularity, and delineate vascular architecture of renal masses [9]. However, CEUS, CECT, and CE-MRI all require the use of contrast agents, which may cause allergic reactions, and their relatively high cost can impose an additional financial burden on patients.

Neovascularization is closely associated with the growth, invasion, metastasis, and recurrence of tumors [10]. Three-dimensional contrast-enhanced ultrasound studies have demonstrated significant differences in vascular morphology and density between benign and malignant renal tumors [11], highlighting the importance of accurately assessing tumor vascularity for differential diagnosis.

With the recent development of advanced ultrasound technologies, it has become possible to noninvasively visualize intratumoral blood flow in detail and with improved accuracy. Superb micro-vascular imaging (SMI) is an advanced wall-filtering algorithm to suppress motion artifacts while preserving low-velocity flow signals, effectively overcoming the limitations of conventional color Doppler flow imaging (CDFI) in detecting slow micro-vessels, without the need for contrast agents [12]. This technique has been increasingly applied in the evaluation and differentiation of tumors in the breast, thyroid, liver, and kidney [12–15]. Compared with CDFI, SMI provides a higher detection rate of vascular signals and Adler grades in both solid and cystic renal lesions, and offers clearer visualization of peripheral and intratumoral vascular branches [15, 16]. Furthermore, the capability of SMI in demonstrating microvascular architecture and its diagnostic performance for solid renal tumors have been reported to be comparable to those of contrast-enhanced ultrasound microflow imaging [17].

Three-dimensional superb micro-vascular imaging (3D-SMI), based on two-dimensional SMI (2D-SMI), enables three-dimensional reconstruction of microvascular structures, allowing visualization of the spatial distribution, morphological features, and perfusion characteristics of microvessels. It provides comprehensive assessment of lesion vasculature from transverse, sagittal, and coronal planes, overcoming the limitation of 2D-SMI, which only offers planar flow information [18]. 3D-SMI can clearly demonstrate the “crab claw-like” branching vessels infiltrating from the periphery toward the center in invasive ductal breast carcinoma, whereas 2D-SMI depicts only partial branches [19]. In addition to visualizing intratumoral vascular architecture stereoscopically, 3D-SMI can be combined with Image-Pro Plus (IPP) software for quantitative analysis. By identifying the plane with the densest vascular signals, color and grayscale pixels within the selected plane are quantified to calculate the vascular index (VI) and vascular area (Area).

In this study, 3D-SMI features of 256 renal tumors were analyzed. Based on 3D vascular visualization, two-dimensional slices were selected for quantitative measurement of VI and Area. The aim was to determine whether 3D-SMI could achieve Vascular Architecture classification and quantitative blood flow assessment without contrast agents, and to evaluate its clinical value in the preoperative diagnosis of renal masses.

## Materials and methods

### Patients

This study was approved by the Ethics Committee of Tianjin Medical University Cancer Institute and Hospital, and written informed consent was obtained from all participants prior to examination. We retrospectively collected data from 512 patients who were first diagnosed with renal masses at our hospital between January 2022 and June 2024 and underwent Gray US, 2D-SMI, and 3D-SMI examinations.

**Fig. 1:**
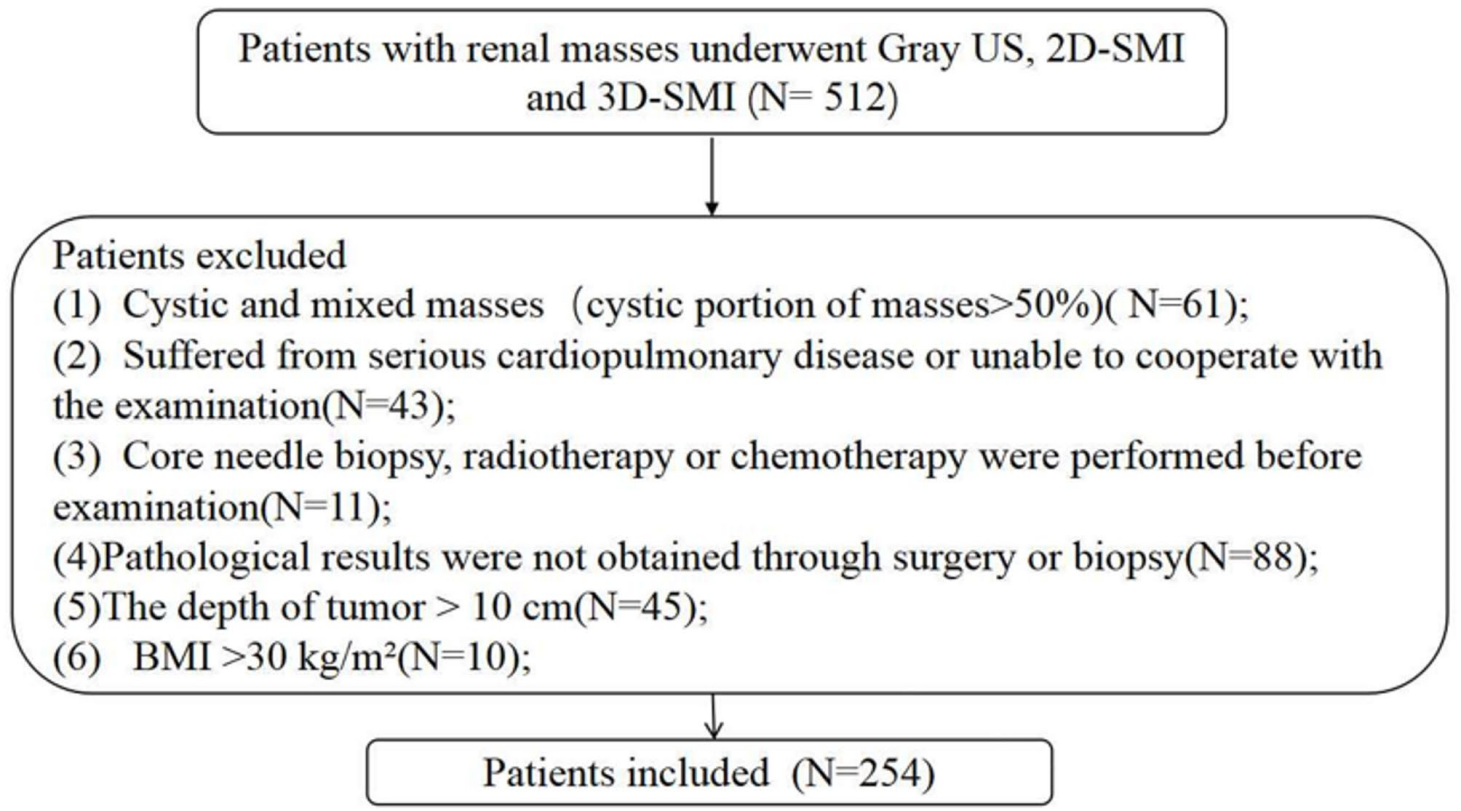
Flow chart of patient selection

Exclusion criteria were as follows: cystic or mixed lesions with > 50% cystic component; severe cardiopulmonary disease or inability to cooperate with the examination; prior biopsy, radiotherapy, or chemotherapy before imaging; lack of pathological confirmation by surgery or biopsy; lesion depth > 10 cm; and body mass index (BMI) > 30 kg/m²(Fig. 1). Ultimately, 254 patients with 256 renal lesions were included, of which 252 were solitary lesions and 2 were ipsilateral double lesions. The mean age of the patients was 56.32 ± 11.9 years.

### Equipment and methods

Gray-scale ultrasound (Gray US), two-dimensional SMI (2D-SMI), and three-dimensional SMI (3D-SMI) examinations were performed using a 1.5–6.0 MHz convex array transducer (PVT-375BT, Aplio500; Toshiba Medical Systems, Tochigi, Japan). All ultrasound scans were conducted by the same sonographer with 12 years of experience in ultrasound diagnosis and 2 years of experience with SMI imaging.

For each lesion, Gray US, 2D-SMI, and 3D-SMI images were obtained. During the examination, the focal zone, scanning depth, and time-gain compensation were adjusted to optimize image quality. Images were captured at end-expiration with breath-holding to minimize respiratory and motion artifacts.

For each lesion, Gray US features were recorded, including: maximum diameter, laterality, location, shape, margin, orientation, echogenicity, internal echo homogeneity, presence of cystic areas, and presence of calcifications.

In SMI, the color velocity scale was set to 1.0–2.0 cm/s, and the color frequency was 5–7 MHz. Vascular features were recorded, including the pattern of blood flow distribution, and semi-quantitative assessment by the Adler grading system [20]. Adler grades were defined as follows: Grade 0 (absent) when no blood flow was detected; Grade I (minimal) when 1–2 pixels contained blood flow; Grade II (moderate) when a main vessel was present in the area and/or 3–4 pixels contained blood flow; and Grade III (marked) when more than 4 blood vessels were detected or the vessels formed an intertwined network.

For 3D-SMI, the transducer was moved slowly and continuously until a complete three-dimensional image was obtained, allowing high-resolution visualization of intratumoral vascular architecture and branching. Based on previous studies of 2D-SMI, three-dimensional power Doppler, and 3D contrast-enhanced ultrasound vascular classification [11, 17, 21] and considering the imaging characteristics of 3D-SMI, vascular architecture were categorized into five types: Type I (absent flow): No detectable blood flow in multiple planes and orientations. Type II (spotty flow): Sparse blood flow signals, with only localized star-like or dot-strip signals observed in some planes.

Type III (minimal flow): Focal intratumoral vascularization, with vessels usually uniform in diameter, simple in branching, and relatively straight. Type IV (peripheral encircling): Vessels surrounding the lesion in an arc, semicircle, or ring shape, with tortuous branches or vascular trees extending from the periphery toward the center, particularly evident in longitudinal views. Type V (extensive flow): Multiple peripheral vessels and numerous intralesional main trunks and branches of varying diameters, forming a complex, tortuous three-dimensional vascular tree or network.

Quantitative analysis of blood flow was performed using Area and VI. A high-resolution three-dimensional vascular map was reconstructed based on 3D-SMI, and the plane with the densest blood flow signals, where both main vessels and branches were clearly visualized, was selected for measurement. The lesion boundary in the selected plane was manually delineated as the region of interest (ROI), and blood flow signals were highlighted in red. Area and VI values were calculated using *Image-Pro Plus* software (version 6.0, Media Cybernetics, Inc., Rockville, MD, USA). Area was defined as the total number of blood flow signal pixels within the ROI. VI was defined as the ratio of blood flow signal pixels to total grayscale pixels within the ROI, multiplied by 100%.

Two radiologists (with 28 years of ultrasound experience and 6 years of SMI experience, and 13 years of ultrasound experience and 3 years of SMI experience, respectively) reviewed the 3D-SMI images of the same renal lesions independently and blindly. They analyzed intratumoral blood flow, assessed Vascular Architecture, and Area and VI values three times for each lesion, with the mean value used for subsequent analysis.

### Statistical methods

Statistical analyses were performed using SPSS version 26.0 (IBM Corp., Armonk, NY, USA). Continuous variables are presented as mean ± standard deviation (SD) or interquartile range (IQR), as appropriate. Interobserver agreement for Vascular Architecture pattern assessment was evaluated using the Kappa statistic, while intraclass correlation coefficients (ICC) were calculated to assess the consistency of Area and VI measurements. Categorical variables were compared using the Chi-square test or Fisher’s exact test, and continuous variables were compared using the Student’s t-test or Mann–Whitney U test, as appropriate. Using pathological results as the reference standard, receiver operating characteristic (ROC) curves were generated to assess the diagnostic performance of Gray-scale US, 2D-SMI, Vascular Architecture, Area, and VI. Differences in the areas under the ROC curves (AUC) were compared using DeLong’s test. A two-sided P value < 0.05 was considered statistically significant.

## Results

### The clinical data, grayscale, and SMI imaging characteristics of the 256 renal masses

A total of 256 renal masses included 70 benign lesions and 186 malignant lesions. The benign lesions included 8 oncocytomas, 3 epithelioid angiomyolipomas (EMAL), 2 metanephric adenomas (MA), and 57 angiomyolipomas (AML). The malignant lesions included 21 papillary renal cell carcinomas (pRCC), 8 chromophobe renal cell carcinomas (chRCC), 4 Xp11.2 translocation/TFE3 gene fusion-related renal cell carcinomas (tRCC), and 153 clear cell renal cell carcinomas (cRCC). The clinical data, grayscale, and SMI imaging characteristics of the 256 renal masses are summarized in Table 1.

**Table 1:** The clinical data, grayscale, and SMI imaging characteristics of the 256 renal masses

		Benign	Malignant	χ^2^/t	*p*
Sex	Male	28	102	4.481	0.034
	Female	42	84		
Max diameter(cm)		4.01 ± 3.21(1.3–12.3)	4.65 ± 2.69(0.6–15.9)	0.437	0.223
Laterality	Left	33	77	0.471	0.493
	Right	37	109		
Location	Upper	29	56	5.452	0.065
	Middle	16	70		
	Lower	25	60		
Depth		4.85 ± 1.84(2.1–8.9)	5.03 ± 1.81(1.9–9.3)	0.003	0.570
Shape	Round/oval	45	97	2.826	0.093
	Irregular	25	91		
Margins	Well	48	94	3.107	0.078
	Poorly	22	92		
Orientation	Inward	16	63	1.080	0.299
	Outward	44	123		
Echo	Hyper	26	49	5.201	0.074
	Iso	23	53		
	Hypo	21	84		
Homogeneity	Homogeneous	28	51	3.206	0.073
	Heterogeneous	42	135		
Cystic component	Absent	62	107	19.522	0.000
	Present	8	73		
Calcification	Absent	65	150	2.497	0.114
	Present	5	38		
distribution	Absent	6	5	7.185	0.066
	Internal	14	38		
	Perilesional	20	36		
	Mixed	29	111		
Adler grade	Grade 0	6	11	25.661	0.001
	Grade I	27	43		
	Grade II	23	45		
	Grade III	14	87		

Comparisons of clinical characteristics and Gray US and 2D-SMI features between benign and malignant renal lesions showed statistically significant differences in patient sex, the presence of cystic components, and Adler grades (*P* < 0.05). However, no significant differences were observed between the two groups in terms of maximal lesion diameter, depth, laterality, location, shape, margin definition, echogenicity, internal homogeneity, presence of calcification, or SMI blood flow distribution (*P* > 0.05).

Interobserver agreement for the classification of Vascular Architecture patterns in renal lesions was excellent, with a Kappa value of 0.803. Quantitative measurements of Area and VI also demonstrated high consistency between the two observers, with ICC values of 0.835 and 0.864, respectively.

### The classification of vascular architecture in the 256 renal tumors using 3D-SMI

**Fig. 2:**
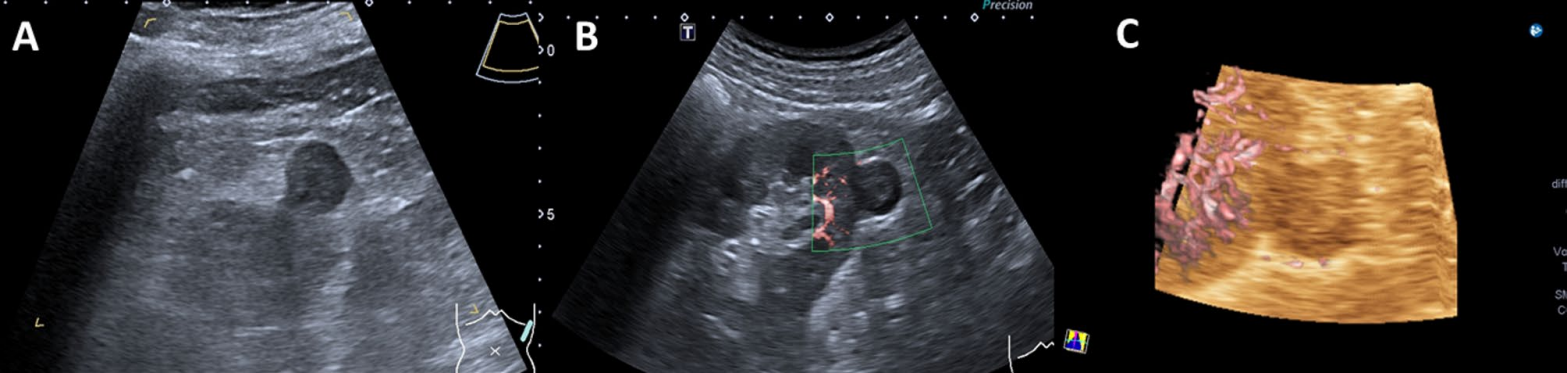
Male patient, 60 years old. **A**: Gray US, a well-defined, solid mass in the lower pole of the left kidney, with a regular shape, homogeneous and hypoechogenicity, a maximum diameter of approximately 1.8 cm. **B**: 2D-SMI, no detectable intralesional blood flow, Adler grade 0. **C**: 3D-SMI, no visible vascular signals on multiple planes, Type I

**Fig. 3:**
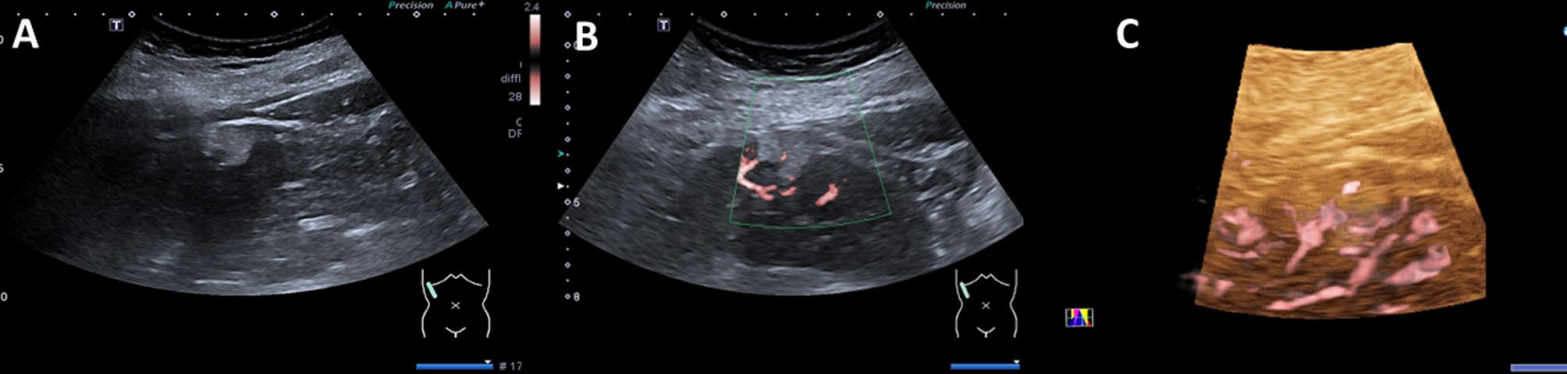
Female patient, 33 years old. **A**: Gray US, solid mass in the middle of the right kidney. with an irregular shape, well marginated, hyperechoic and heterogeneous, a maximum diameter of approximately 2.2 cm. **B**: 2D-SMI, punctate and linear blood flow, Adler grade I. **C**: 3D-SMI, multiplanar observation reveals punctate and linear vascular signals around the periphery of the lesion, Type II

**Fig. 4:**
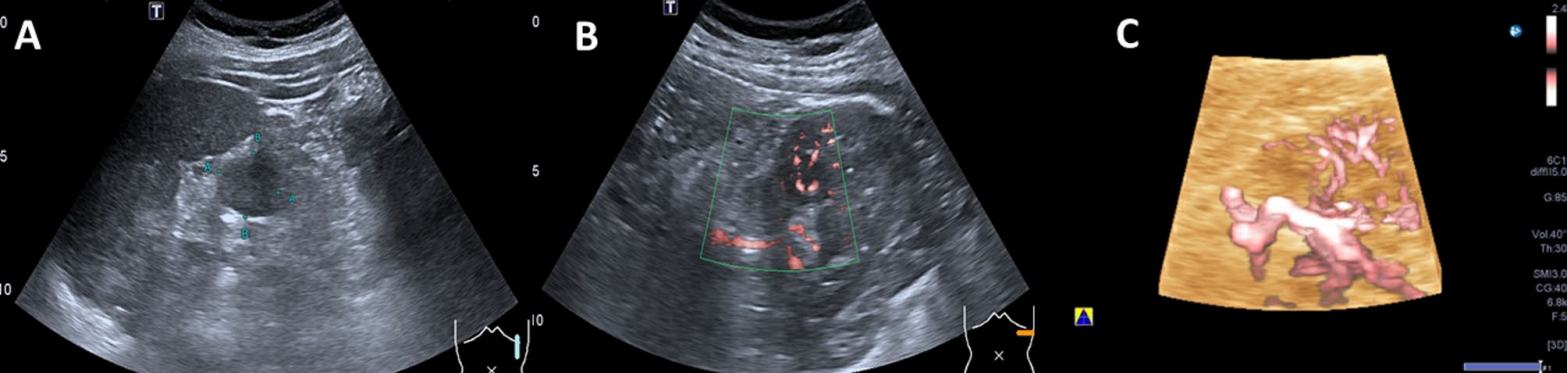
Female patient, 49 years old. **A**: Gray US, solid mass in the upper pole of the left kidney, with an irregular shape, poorly marginated, hypoechoic and heterogeneous, a maximum diameter of approximately 1.9 cm. **B**: 2D-SMI, punctate blood flow signals are observed around the periphery of the lesion, Adler grade II. **C**: 3D-SMI, peripheral vessels are extending toward the center, penetrating vessels run relatively straight with simple branching patterns, Type III

**Fig. 5:**
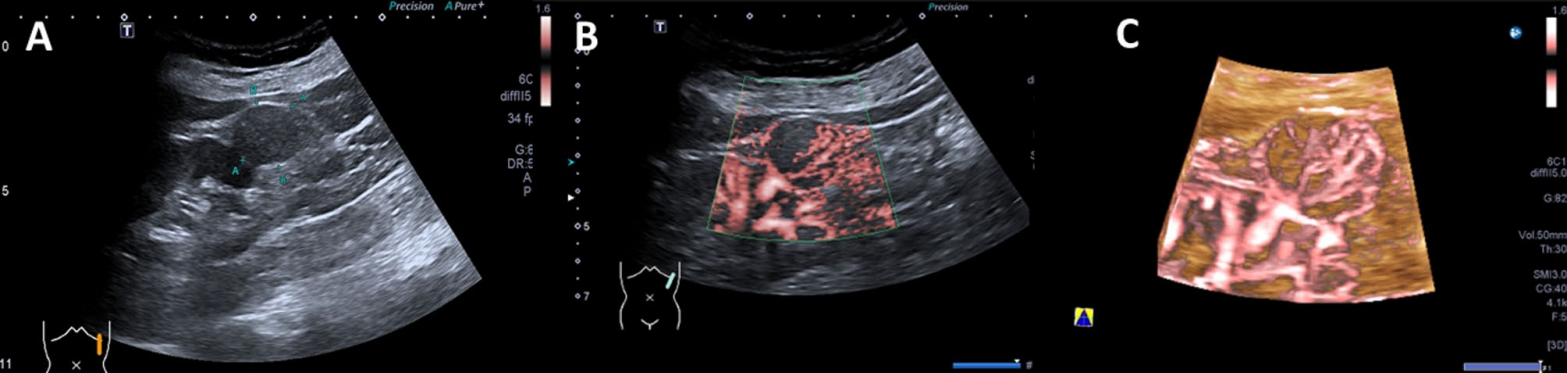
Female patient, 55 years old. **A**: Gray US, solid mass in the lower pole of the left kidney. with a regular shape, well marginated, hypoechoic and heterogeneous, a maximum diameter of approximately 2.7 cm. **B**: 2D-SMI, abundant blood flow signals are detected both center and around the lesion, Adler grade III. **C**: 3D-SMI, circumferential peripheral vessels encircle the lesion, with tortuous vascular branches extending from the periphery toward the center, Type IV

**Fig. 6:**
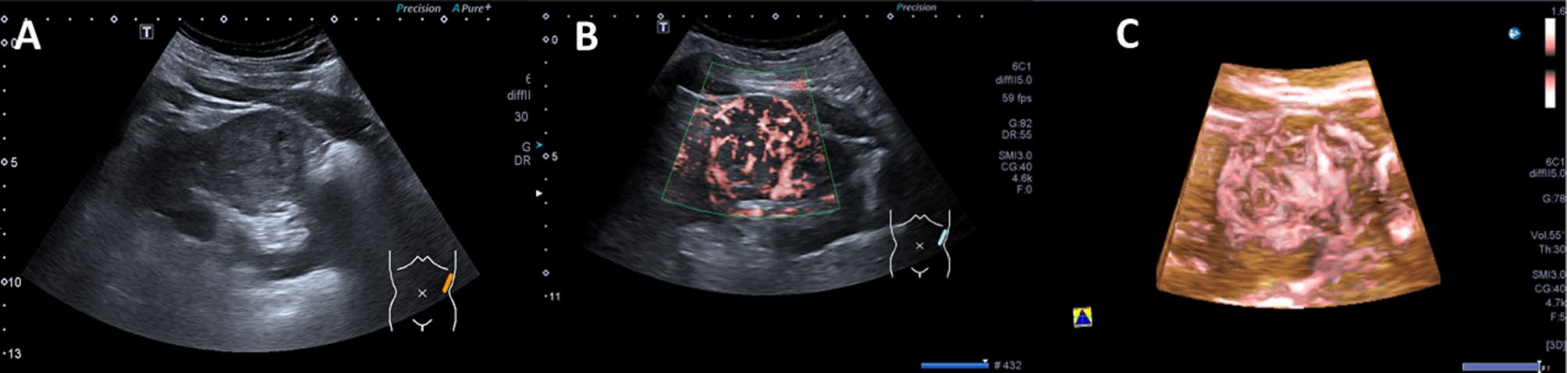
Male patient, 70 years old. **A**: Gray US, solid mass in the middle of the left kidney. with an irregular shape, poorly marginated, hypoechoic and heterogeneous, and small cystic areas, a maximum diameter of approximately 4.6 cm. **B**: 2D-SMI, abundant blood flow signals are detected both within and around the lesion, Adler grade III. **C**: 3D-SMI, multiple vessels of varying calibers form complex vascular trees or networks throughout the lesion, with tortuous and irregular courses, Type V

Comparison of 3D-SMI Vascular Architecture revealed a statistically significant difference between benign and malignant renal tumors (χ² = 39.178, *P* < 0.001). A total of 17 lesions showed no detectable blood flow signal and were classified as Type I, including six benign and eleven malignant lesions. Benign tumors were predominantly characterized by Type II and Type III vascular patterns, whereas malignant tumors mainly exhibited Type IV and Type V patterns, with Type V being the most common subtype in the malignant group (38.7%). The distribution of 3D-SMI vascular patterns for all 256 renal lesions is summarized in Table 2, and representative images of each vascular pattern are shown in Figs. 2, 3, 4, 5 and 6.

**Table 2:** The classification of vascular architecture in the 256 renal tumors using 3D-SMI(n)using smart 3D-SM

	Type I	Type II	Type III	Type IV	Type V	χ^2^	*P*
Benign	6	13	35	9	7	39.178	0.000
Malignant	11	14	39	50	72		

### Vascular architecture, Area, and VI of renal masses with different pathological types assessed by smart 3D-SMI

**Fig. 7:**
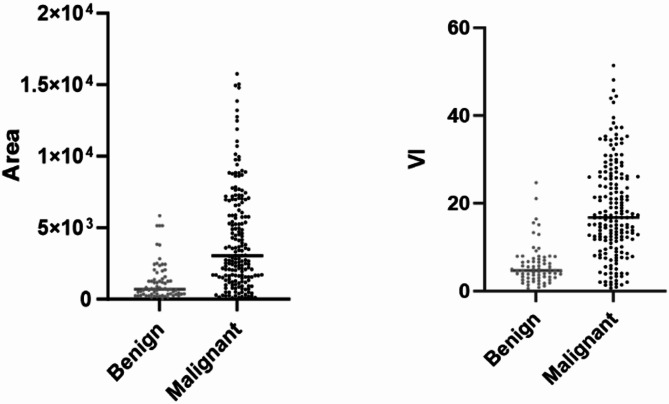
The distribution of Area and VI values for benign and malignant renal tumors

This study quantitatively analyzed the blood flow information of renal tumors using the blood flow pixel Area and the Vascular Index (VI) for both benign and malignant lesions. The Area and VI values for 256 renal masses are presented in Fig. 5. Benign Renal Tumors: The mean Area was 945.87 ± 568.26 (range: 68–3125). The Area was divided into quartiles as follows: Q1 ≤ 206, Q2 > 206 and ≤ 405, Q3 > 405 and ≤ 1259, and Q4 > 1259. The mean VI was 5.93 ± 4.95 (range: 0.23–24.73), with quartiles defined as: Q1 ≤ 2.81, Q2 > 2.81 and ≤ 4.31, Q3 > 4.31 and ≤ 7.38, and Q4 > 7.38. Malignant Renal Tumors: The mean Area was 3694.53 ± 2612.38 (range: 93–9965). The Area was divided into quartiles as follows: Q1 ≤ 1469, Q2 > 1469 and ≤ 3076, Q3 > 3076 and ≤ 6591, and Q4 > 6591. The mean VI was 18.21 ± 10.83 (range: 0.69–48.13), with quartiles defined as: Q1 ≤ 9.43, Q2 > 9.43 and ≤ 16.71, Q3 > 16.71 and ≤ 25.99, and Q4 > 25.99. The distribution of Area and VI values for benign and malignant renal tumors is shown in Fig. 7. Statistical analysis revealed that the Area and VI values of malignant renal tumors were significantly higher than those of benign lesions (F = 37.450, *P* < 0.000; F = 50.060, *P* < 0.000).

### Receiver operating characteristic (ROC) curve analysis for malignant renal masses

**Fig. 8:**
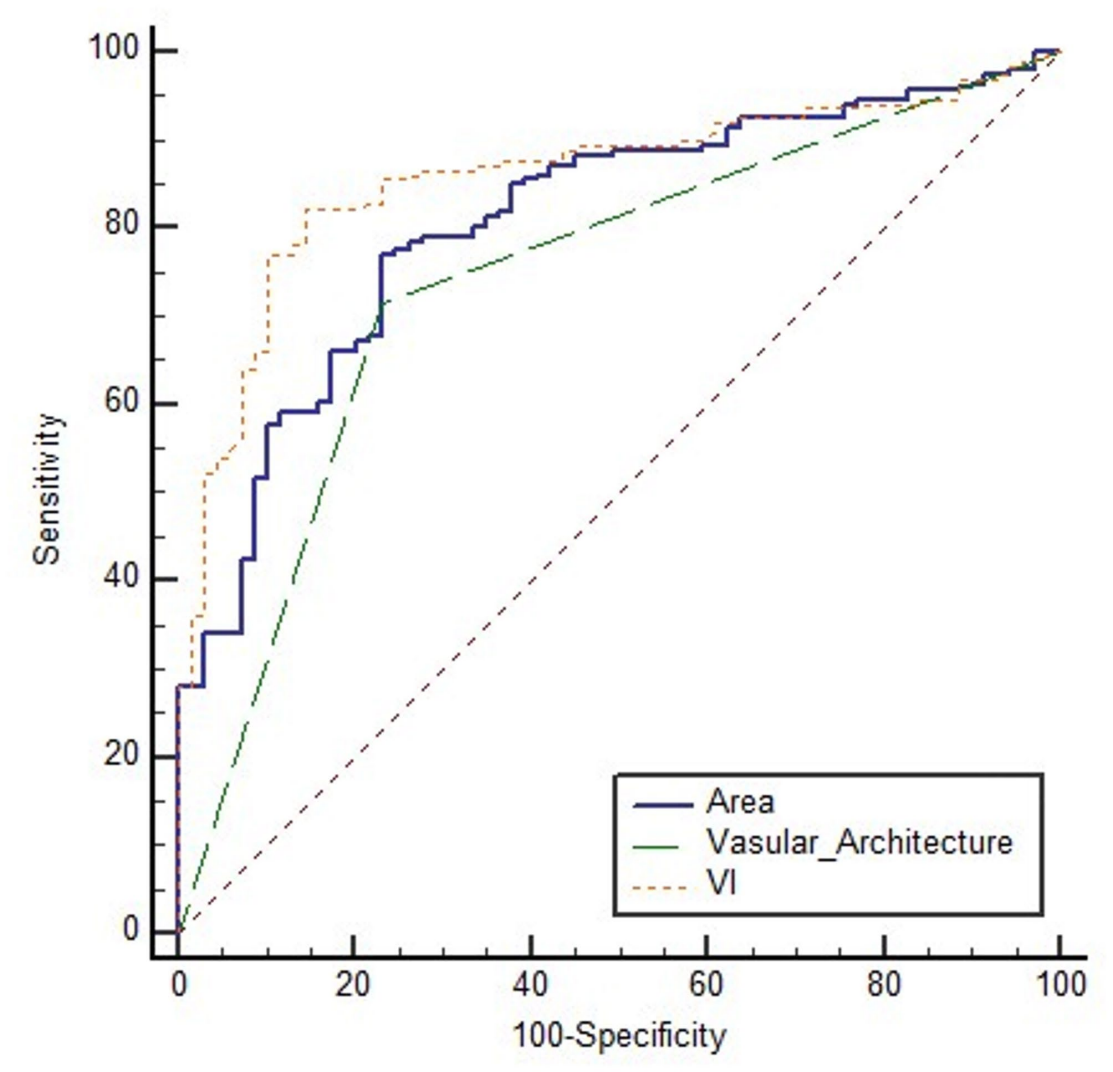
ROC curve of Gray US, 2D-SMI, Vascular Architecture, Area, and VI in differentiating benign from malignant renal masses

The diagnostic performance of Gray US, 2D-SMI, Vascular Architecture, Area, and VI in differentiating benign from malignant renal lesions was assessed using receiver operating characteristic (ROC) curve analysis (Fig. 8). The sensitivity, specificity, accuracy, area under the ROC curve (AUC), Youden index (J), and 95% confidence intervals for each parameter are summarized in Table 3. According to the Z-test for AUC comparison, 2D-SMI, Vascular Architecture, Area, and VI exhibited significantly better diagnostic performance than Gray US (*p* < 0.05). Vascular Architecture and VI demonstrated superior diagnostic efficacy compared with 2D-SMI and vascular area (*p* < 0.05). An Area greater than 1430 was determined as the optimal cutoff for identifying malignant lesions, with a sensitivity of 76.88% and specificity of 76.81%. Similarly, a VI greater than 8.19 was identified as the optimal threshold for malignancy, achieving a sensitivity of 82.26% and specificity of 85.51%.

**Table 3:** Performance of different diagnostic for methods between benign and malignant renal masses

	Sensitivity,%	Specificity,%	Accuracy, %	AUC	Youden index J	95% Confidence interval
Gray US	76.88	66.67	73.8	0.718	0.435	0.658–0.772
2D-SMI	79.03	71.01	76.6	0.750	0.500	0.692–0.802
Vascular Architecture	82.80	79.71	81.64	0.813	0.6251	0.759–0.859
Area	77.88	76.81	76.17	0.807	0.5369	0.753–0.854
VI	82.26	85.51	83.20	0.859	0.6777	0.810–0.900

### Vascular architecture, area, and VI with different pathological types of renal masses by 3D-SMI

**Fig. 9:**
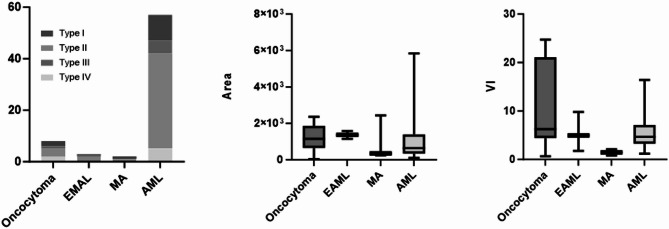
The distribution of Vascular Architecture, Area and VI in different pathological types benign tumors

**Fig. 10:**
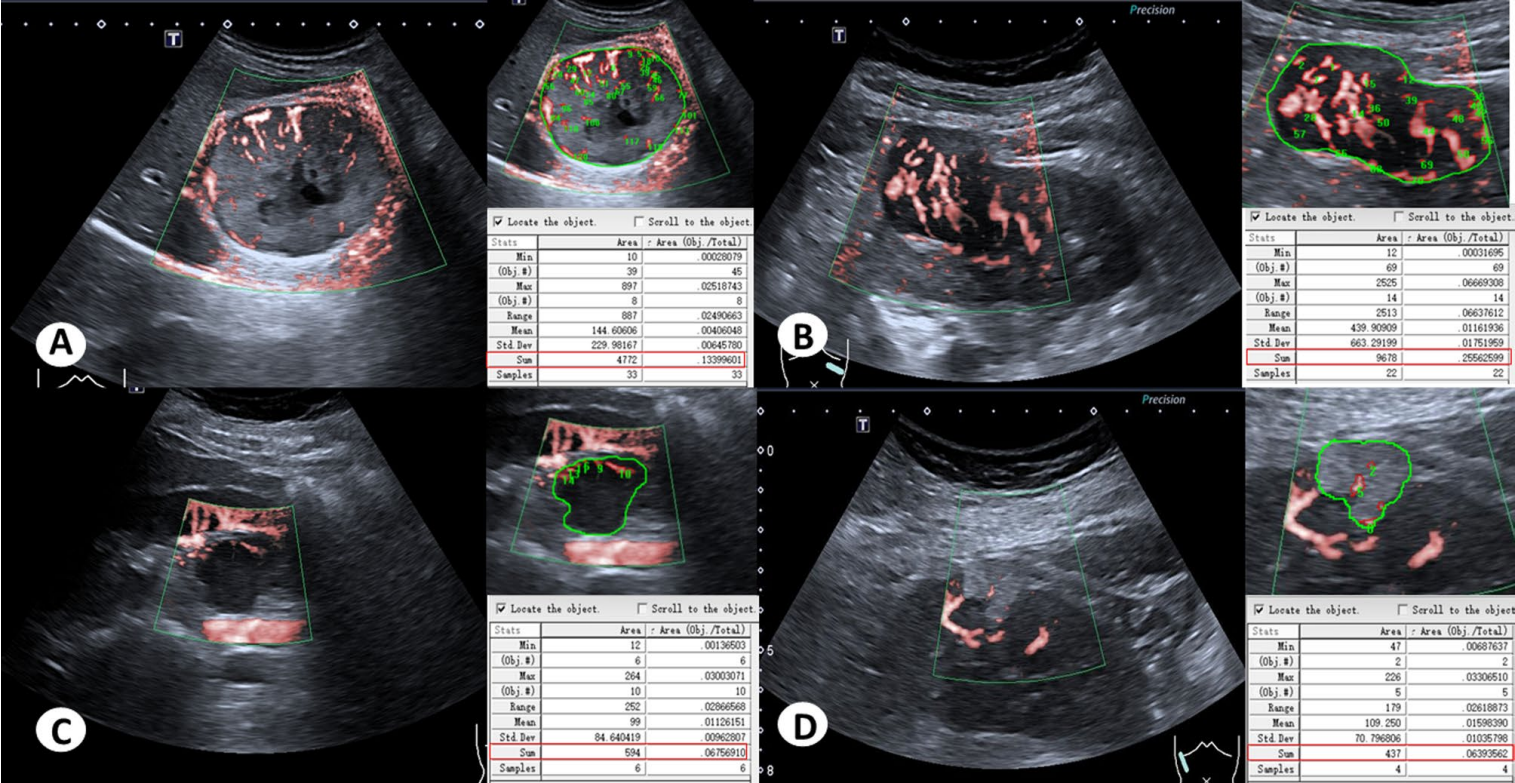
Area and VI of Benign renal masses with different pathological types. (**A**) Oncocytoma, Area: 4772, VI: 13.39. (**B**) EMAL, Area: 9678, VI: 25.56. (**C**) MA, Area: 574, VI: 6.76. (**D**) AML, Area: 437, VI: 6.39

**Fig. 11:**
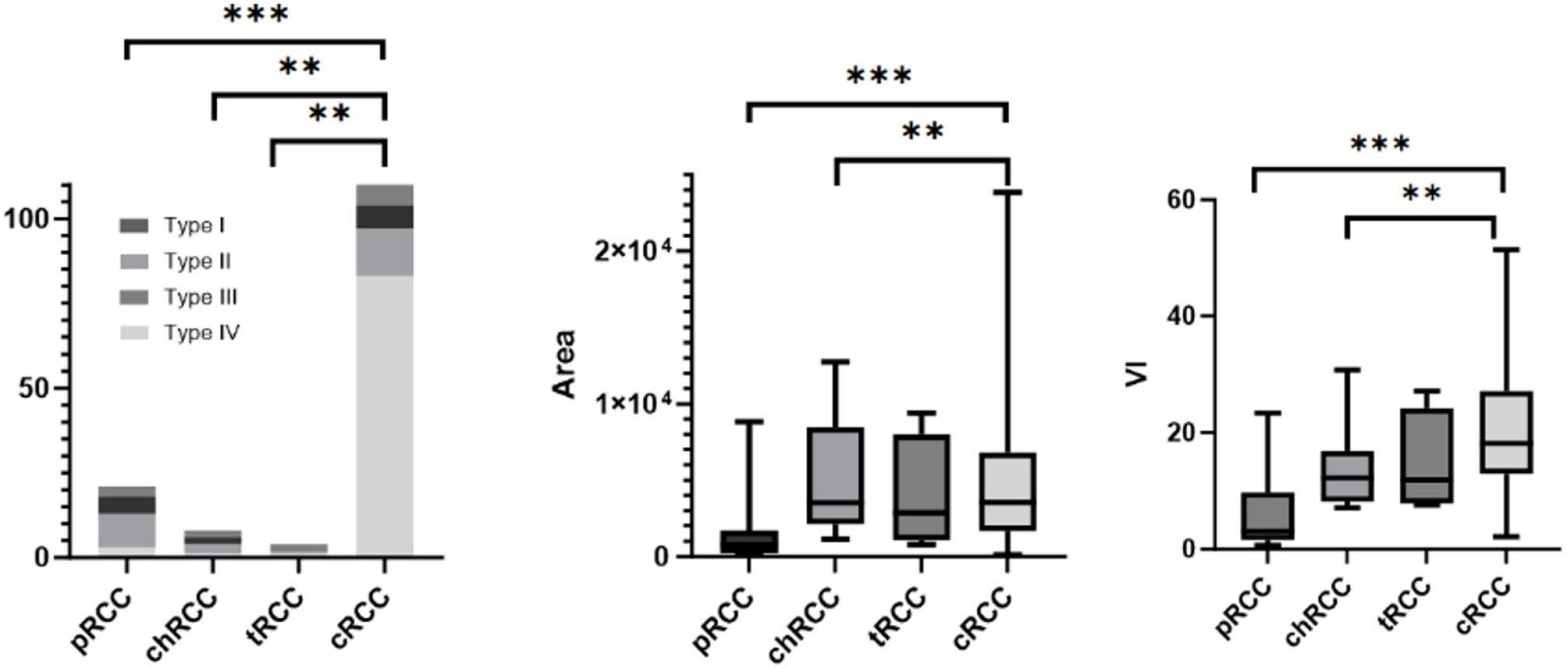
The distribution of Vascular Architecture, Area and VI in different pathological types malignant tumors

**Fig. 12:**
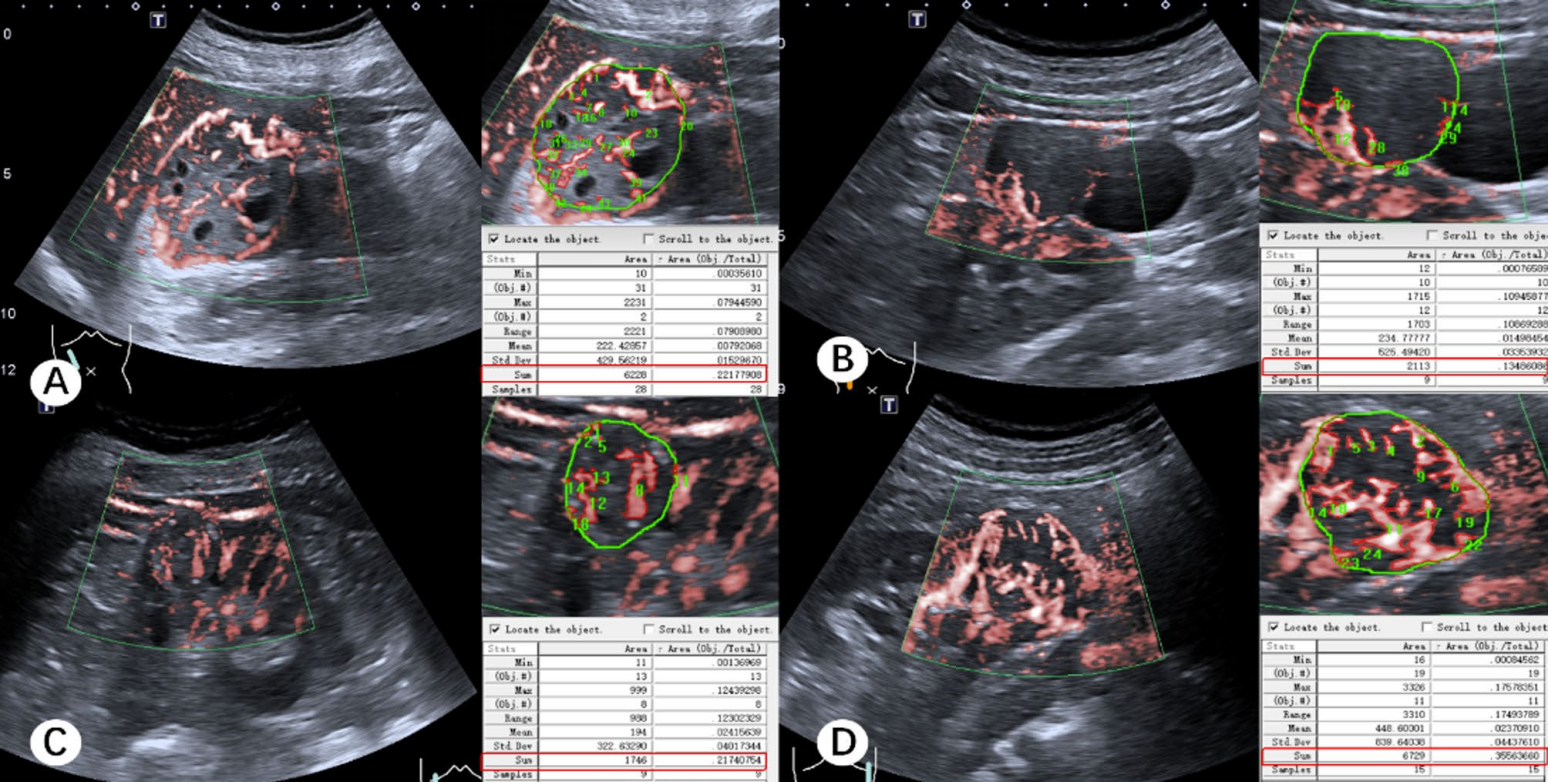
Area and VI of malignant renal masses with different pathological types. (**A**) pRCC, Area: 6228, VI: 22.18. (**B**) chRCC, Area: 2113, VI: 13.49. (**C**) tRCC, Area: 1746, VI: 21.75. (**D**) ccRCC, Area: 6729, VI: 35.56

The 3D-SMI Vascular Architecture, Area, and VI of renal lesions with different pathological types are summarized in Table 4. Among benign lesions, no significant differences were observed in 3D-SMI Vascular Architecture or Area across different pathological subtypes (*P* > 0.05). However, the VI of oncocytomas was significantly higher than that of EAML, MA, and AML (*P* < 0.01; Figs. 9 and 10). For malignant lesions, the vascular architecture of ccRCC differed significantly from that of pRCC, chRCC, and tRCC (*P* < 0.01). Additionally, the Area and VI of ccRCC were significantly higher than those of pRCC and chRCC (*P* < 0.05), whereas no significant differences were observed when compared with tRCC (*P* > 0.05; Figs. 11 and 12).

**Table 4:** Vascular architecture, Area, and VI of renal masses with different pathological types assessed by 3D-SMI

		Oncocytoma	EAML	MA	AML	pRCC	chRCC	tRCC	cRCC
Vascular Architecture	I	1	0	0	5	1	1	0	9
	II	2	0	1	10	4	1	0	7
	III	3	2	1	36	10	3	1	14
	IV	1	1	0	3	3	2	2	46
	V	1	0	0	3	3	1	1	77
Area		1140.00 ± 778.12 (34 ~ 2364)	1014.00 ± 1234.13 (251 ~ 2438)	330.00 ± 384.74 (58 ~ 602)	958.11 ± 1440.29 (13 ~ 5844)	1598.23 ± 2616.00 (93 ~ 8837)	2725.03 ± 1751.48 (1131 ~ 4600)	3981.75 ± 3225.51 (779 ~ 9406)	4761.73 ± 4115.61 (110 ~ 23851)
VI		10.72 ± 9.15 (0.63 ~ 24.73)	5.50 ± 4.04 (1.76 ~ 9.78)	4.35 ± 4.00 (0.81 ~ 7.89)	4.31 ± 4.28 (0.58 ~ 16.42)	6.05 ± 6.84 (0.69 ~ 23.42)	14.05 ± 4.75 (8.56 ~ 16.89)	14.63 ± 9.01 (7.6 ~ 27.16)	20.52 ± 10.55 (2.1 ~ 51.4)

## Discussion

Tumor growth is closely associated with neovascularization [22] As tumors enlarge, branches extend internally and peripherally, forming abundant microvessels that infiltrate surrounding tissues, and may also facilitate local invasion and distant metastasis [23]. Therefore, the morphology and distribution of intratumoral vessels are closely related to tumor characteristics and serve as important indicators for differentiating benign and malignant tumors [24]. Conventional methods, such as color Doppler ultrasound, can evaluate intralesional blood flow but have limited sensitivity for detecting low-velocity flow [25]. SMI preserves low-velocity blood flow signals, enabling rapid, convenient, and noninvasive assessment of tumor perfusion [26]. While 2D-SMI allows observation and evaluation of blood flow in a single imaging plane, 3D-SMI provides a three-dimensional view of vascular spatial course, branching, and interrelationships, revealing microvascular details and permitting assessment of complex vascular structures ^[19]^. By observing tumors from multiple angles and planes with 3D-SMI, the plane with the richest blood flow can be identified, allowing more accurate calculation of parameters such as Area and VI, thus providing a noninvasive and reliable quantitative basis for clinical diagnosis.

Basing on previous 2D-SMI studies [17], research on 3D power Doppler and contrast-enhanced 3D ultrasound vascular classification [11, 21], this study proposes, for the first time, a 3D-SMI based vascular architecture classification of renal tumors through the distribution, morphology, branching complexity, and spatial course of intratumoral and peritumoral vessels. Tumor vascular architecture was categorized into five types.

In this study, benign renal lesions predominantly exhibited Type II or III, whereas malignant lesions were mainly Type IV or V, consistent with previous reports [17]. This pattern is primarily related to the rich vascular supply and active neovascularization in malignant renal tumors [27]. Rapid growth of malignant tumors necessitates increased oxygen and nutrient supply, which is achieved by elevating vessel density and blood flow. Tumor cells secrete pro-angiogenic factors such as VEGF (vascular endothelial growth factor) and bFGF (basic fibroblast growth factor) to stimulate endothelial cell proliferation, resulting in markedly increased intratumoral perfusion [28]. neovascularizations grow in a disorganized manner, with chaotic and tortuous courses [29], consistent with the 3D-SMI findings. Additionally, as malignant renal tumors infiltrate surrounding tissue, they compress adjacent normal parenchyma and vessels, forming a ring of relatively abundant peripheral vessels. A pseudocapsule composed of fibrous tissue and vessels often surrounds renal carcinoma [30], which on imaging manifests as a characteristic ring- or semicircle-shaped vascular distribution, resembling a “corolla-like” pattern [31], corresponding to Type IV in this study.

Previous studies [32] evaluating renal tumor blood flow predominantly relied on Adler grading, which is relatively simple and assesses blood flow based primarily on the number and location of vessels. In Adler grading, more than four vessels or the presence of a vascular network is classified as Grade III, which does not fully capture the complex vascular architecture. In the present study, Area and VI were introduced as quantitative indicators of blood flow.

Using 3D-SMI, the plane with the richest blood flow was selected, and Image Pro Plus (IPP) software was applied to calculate the total number of pixels representing color Doppler signals within the ROI (Area) and the ratio of color pixels to grayscale pixels within the tumor (VI), directly reflecting the richness of intratumoral blood flow and vascular density. Compared with benign lesions, malignant renal tumors exhibited significantly higher Area and VI values, consistent with previous studies and the biological characteristics of benign and malignant renal tumors [33]. In this study, the VI values were slightly higher than those reported previously [33] (benign: 5.93 ± 4.95 vs. 4.30 ± 4.27; malignant: 18.21 ± 10.83 vs. 14.95 ± 10.94). This difference may be attributable to the earlier studies selecting the ROI from a single plane, potentially missing portions of microvascular signals. By contrast, our study utilized 3D reconstruction to identify the plane with the most abundant blood flow, providing a more comprehensive visualization of fine vessels and increasing the detection of tortuous branching in malignant tumors, resulting in higher VI measurements.

ROC curve analysis demonstrated that the diagnostic performance of 2D-SMI, 3D-SMI, Area, and VI all surpassed that of Gray US. The diagnostic performance of Area did not differ significantly from that of 2D-SMI, whereas VI exhibited the highest diagnostic accuracy. The VI is calculated as the ratio of color signal pixels to grayscale pixels within the tumor, reflecting vascular density. This approach considers not only the absolute amount of blood flow signals but also the tumor volume, providing a more comprehensive assessment of the relative richness of vessels per unit volume. In contrast, Area represents the absolute area of blood flow signals without accounting for tumor size. Therefore, in cases with heterogeneous vascular distribution, VI offers a distinct advantage.

Analysis of 3D-SMI across different pathological types of renal malignant masses revealed distinct Vascular Architectures, Area, and VI. The ccRCC predominantly displayed type IV and V, characterized by abundant peripheral blood flow with tortuous, irregular branching extending from the periphery toward the tumor center. In contrast, pRCC and chRCC mainly exhibited type II and III, with sparse local vascular implantation, relatively simple branching, and more uniform vessel diameters. The Area and VI values of ccRCC were comparable to those of tRCC and significantly higher than those of pRCC and chRCC. Correspondingly, CT attenuation values of ccRCC during the corticomedullary, nephrographic, and excretory phases are higher than those of pRCC and chRCC [34]. The histological architecture of pRCC typically forms papillary or tubular structures, whereas chRCC cells are often polygonal with minimal stroma and densely packed cells. Their tumor stroma contains abundant fibrous tissue, which inhibits angiogenesis, resulting in sparse intratumoral vasculature [35] and relatively low expression of angiogenic factors such as VEGF, indicating limited neovascularization activity [36]. These biological characteristics render pRCC and chRCC hypovascular, which serves as an important distinguishing feature from ccRCC.

Among benign renal tumors, oncocytomas exhibited significantly higher VI values than other benign types. A single-center study reported that oncocytomas show marked enhancement during the arterial phase on contrast-enhanced ultrasound, with clearer tumor borders post-contrast, although no specific features allow differentiation from RCC [37]. On contrast-enhanced CT, oncocytomas demonstrate strong cortical phase enhancement, slow washout in the parenchymal phase, and central scars without enhancement in some larger tumors [38]. Histologically, these tumors consist of well-differentiated eosinophilic cells arranged in nests, with stroma rich in capillaries [39]. Findings from CEUS, CECT, and pathology consistently indicate that oncocytomas are hypervascular, which is consistent with the elevated VI values observed in this study.

This study is the first to propose a classification system for renal mass vascular architecture based on 3D-SMI and to introduce Area and VI as quantitative parameters for assessing blood flow richness and vascular density, thereby enhancing the objectivity and reproducibility of diagnosis. Nevertheless, several limitations should be acknowledged. Operator skill and experience may still exert subjective influence on image quality and measurements, highlighting the need for further standardization of scanning protocols. 3D-SMI does not provide dynamic tissue perfusion information. Patient BMI, as well as tumor location and depth, can affect image quality, and in obese patients or for deeply located tumors, complementary modalities such as CEUS may still be necessary. Furthermore, 3D-SMI cannot currently differentiate arterial from venous flow, which may limit assessment of tumor vascular supply patterns. As a non-invasive ultrasonographic technique, future studies should focus on optimizing image analysis algorithms to reduce operator dependence and integrating multimodal imaging approaches to improve diagnostic accuracy and reliability.

3D-SMI enables multi-angle, multi-plane, three-dimensional visualization of the microvascular network within renal masses without the use of contrast agents. By selecting the plane with the most abundant blood flow from the 3D-SMI dataset and combining it with quantitative analysis using Area and VI, this approach provides an objective and quantitative method for the non-invasive preoperative characterization of renal tumors. It has the potential to reduce misdiagnosis and missed diagnoses, thereby supporting more informed clinical decision-making.

## Data Availability

The datasets used during the current study are available from the corresponding author on reasonable request.
